# Cancer

**Published:** 1881-03

**Authors:** 


### CANCER.

Cancer is comparatively a rare disease; but since the aggregate of suffering is greater among those who imagine that they have or are threatened with cancer, than among those who really have the disease, its symptoms should be known by all.

Cancer on the surface of the body begins with a small sore or pimple, that has but little sensibility at first but quickly increases in size and sensitiveness until it becomes very painful.

The character of the pain is peculiar. At first it is sharp and quick at intervals; then there is a painful clawing sensation, as if an eagle were tearing the flesh, and this is followed by a dull pain. In the meantime ulceration is established, and the disease rapidly extends and destroys. Its march is steadily onward. Therefore, when a suspicious sore is arrested in its progress and heals up, or even partially heals and then breaks out again, it is not cancer.

Cancer sometimes attacks the glands, or some deep-seated portion of the body. Here again, fortunately, but few of the suspicious swellings are cancerous, and most of them remain stationary for years, or perhaps during life, while some disappear spontaneously.

### DON'T LET THE FIRE GO OUT.

Curtis Andrews, living in the fourth district of Carolina County, is now eighty-two years old. His wife is nearly the same age, and they have lived together for sixty years. Their life has been plain and laborious, but their faces wear a look of smiling content that draws kindly feeling toward them. When asked the secret of his happiness, Andrews replied: "Well, sir, I have always noticed that there is more trouble between man and wife over making fire in the morning than anything else. If they can get along smoothly about that, everything else is smooth.

"My wife and I went to housekeeping together in our log cabin fifty years ago. We've only got one fireplace, but that's a big one. When we moved in I said to her: 'Sally, I'll make the fire and I'll tend to it.' I made that fire, and it's been burning ever since. For nigh fifty years I've fixed it up in the morning. I've never had any matches in the house, and there are never any sulphur smells in the household. While that fire burns, sir, there is peace in Curtis Andrews' house."

THE great rule of moral conduct is, next to God, to respect Time.

